# Hybrid Platforms of Silicon Nanowires and Carbon Nanotubes in an Ionic Liquid Bucky Gel

**DOI:** 10.3390/molecules27144412

**Published:** 2022-07-09

**Authors:** Maria José Lo Faro, Antonio Alessio Leonardi, Dario Morganti, Sabrina Conoci, Barbara Fazio, Alessia Irrera

**Affiliations:** 1 Department of Physics and Astronomy, University of Catania, Via Santa Sofia 64, 95123 Catania, Italy; mariajose.lofaro@dfa.unict.it (M.J.L.F.); antonio.leonardi@dfa.unict.it (A.A.L.); 2 CNR-IMM UoS Catania, Via Santa Sofia 64, 95123 Catania, Italy; sabrina.conoci@unime.it; 3 Department of Chemical, Biological, Pharmaceutical, and Environmental Sciences, University of Messina, Viale Ferdinando Stagno D’Alcontres 5, 98166 Messina, Italy; dario.morg@hotmail.it; 4 URT LAB SENS, Beyond Nano—CNR, Viale Ferdinando Stagno D’Alcontres 5, 98166 Messina, Italy; 5 CNR-IPCF, Istituto per i Processi Chimico-Fisici, Viale F. Stagno D’Alcontres 37, 98158 Messina, Italy

**Keywords:** silicon nanowires, ionic liquids, carbon nanotubes, bucky gel, photovoltaic, photocurrent

## Abstract

Silicon nanowires (NWs) are appealing building blocks for low-cost novel concept devices with improved performances. In this research paper, we realized a hybrid platform combining an array of vertically oriented Si NWs with different types of bucky gels, obtained from carbon nanotubes (CNT) dispersed into an ionic liquid (IL) matrix. Three types of CNT bucky gels were obtained from imidazolium-based ionic liquids (BMIM-I, BIMI-BF_4_, and BMIM-Tf_2_N) and semiconductive CNTs, whose structural and optical responses to the hybrid platforms were analyzed and compared. We investigated the electrical response of the IL-CNT/NW hybrid junctions in dark and under illumination for each platform and its correlation to the ionic liquid characteristics and charge mobility. The reported results confirm the attractiveness of such IL-CNT/NW hybrid platforms as novel light-responsive materials for photovoltaic applications. In particular, our best performing cell reported a short-circuit current density of 5.6 mA/cm^2^ and an open-circuit voltage of 0.53 V.

## 1. Introduction

Today, silicon nanowires (Si NWs) and carbon nanotubes (CNTs) are considered some of the most promising nanostructures, offering enormous potential for a wide range of applications [1,2,3]. Furthermore, both Si NWs and CNTs demonstrate enhanced optical absorption and electrical conduction; hence, the possibility of creating coaxial junctions based on these two nanostructures is of great interest for energy production devices [4,5].

In fact, Si NWs are strategic systems for a plethora of applications in photonics [6], microelectronics [7,8], photovoltaics [9], and sensing [10] due to their one-dimensional features and physical-chemical properties. Regardless, surmounting the limits of modern devices requires the efforts of the international scientific community for the realization of radial junctions based on this material [11,12]. Indeed, the production of Si NW coaxial p-n junctions by using several growth techniques has been reported in the literature and some of their major limitations arise from the variability of the doping profile, structure quality, and low uniformity [7,13]. Conversely, the use of lithographic processes with nanoscale features involves extremely high costs, a long production time, and extremely small processable areas, resulting in low outputs not suitable for industrial applications and market targets [14,15]. In this study, we created hybrid radial junctions based on a core of Si NWs embedded into a bucky gel composed of CNTs well dispersed in an ionic liquid (IL) matrix. The choice of carbon nanotubes as light-responsive materials for the hybrid Si NWs device was due to their very interesting properties arising from their one-dimensional structure [16]. The electronic properties of CNTs are due to the electrons of the 2p_z_ orbitals, which do not participate in any bond and remain delocalized, allowing good electrical conduction [17]. S. Iijima at the NEC Research Laboratory first observed in 1991 one-dimensional carbon allotropes as multi-walled CNTs (MWCNTs) [18], and later in 1993 single-walled CNTs (SWCNTs) [19]. Since then, a considerable amount of growth techniques have been available for nanotube production [20], such as arc discharge [21], laser ablation [22], high-pressure carbon monoxide [23], and chemical vapor deposition [24,25]. Most of these processes take place in a vacuum or with the aid of a process gas, synthesizing large quantities of nanotubes for both research applications and commercialization [26,27]. Similar to diamond, graphite, and fullerene, nanotubes are particular 1D allotropes of carbon, with a peculiar electronic structure uniquely identified by a pair of natural indexes (n, m) and the **a**_1_ and **a**_2_ cell vectors, which determine the chiral vector **C** = n**a**_1_ + m**a**_2_, around which the graphene sheet wraps around itself. SWCNTs are an important variety of carbon nanotubes because most of their properties change significantly with (n, m) values such as their band-gap (from zero to about 2 eV) and their electrical conductivity from metallic to semiconductor behavior. Most single-walled nanotubes have a diameter of nearly 1 nanometer, with a tube length that can be many millions of times longer, exhibiting a high length-to-diameter ratio significantly larger than other materials [28].

For these reasons, SWCNTs are excellent conductors with physical properties of great importance for nanotechnology, electronics, optics, and other technological fields [16,29,30,31]. Furthermore, CNTs are also efficient absorbers able to trap light across the entire solar spectrum and therefore are considered strategic for future photovoltaic applications [32,33]. Despite their advantages in photovoltaics, CNT use is hampered by the π-π interactions among different nearby CNTs, leading to the formation of a 3D network of bundled wires. When bundled, the CNT optical properties undergo quenching due to charge transfer and their electric conduction suffers from charge trapping and recombinations [34]. Hence, for the practical use of CNTs in real devices, their debundling in suitable solvents represents a critical aspect [35]. Bonaccorso et al. reported the role of different bile salts, such as sodium cholate (SC), sodium deoxycholate (SDC), and sodium taurodeoxycholate (TDC), which are more effective for CNT debundling and individualization compared to linear chain surfactants such as sodium dodecylbenzene sulfonate (SDBS) and sodium dodecyl sulfate (SDS) [36]. Micelles, polymers, and other solvents which attenuate the mutual nanotube electrostatic interactions can be also used for CNT debundling [37,38,39].

More recently, the race toward innovative technologies promoted the development of green chemistry to minimize the environmental risks from anthropogenic activity with new synthesis methodologies designed to reduce the waste products into the biosphere using non-contaminating raw materials [40]. Green chemistry has opened up new frontiers for eco-compatible regents compared to traditional organic solvents. Among these systems, supercritical fluids and a new class of solvents, such as ionic liquids (ILs), have wide industrial applications [41]. ILs are pure organic salts consisting of a cation–anion pair, which unlike conventional Molten salts (T_melt_ = 800 °C), have melting temperatures well below 100 °C. In particular, room temperature ionic liquids (RTIL) possess a negligible vapor pressure, and a very high thermal stability from −50 to 100 °C, combined with a considerable ionic conductivity and electrochemical stability in the range from 4.5 to 6 V [42]. ILs also present a controllable viscosity and are adopted as non-flammable solvents for both organic and inorganic compounds. The low melting temperatures are the result of the asymmetric chemical composition of the RTILs, generally presenting a large organic cation and smaller inorganic counterparts. This structural asymmetry of ILs reduces the lattice energy, hence the melting point of the resulting ion medium. The properties of ILs vary depending on the nature of the cation and anion combinations, giving rise to a large number of ILs (about 10^18^), of which only a few are commercially available. Some of the most common ILs used for experimental purposes are based on the imidazole anion chain [43]. Their peculiar properties allow for applications in various fields, such as organic synthesis, catalysis, electrochemistry, analytical chemistry, and nanotechnology. The applications in electrochemical devices include solar cells [44,45], Li-IL batteries [46], capacitors [47], and fuel cells [48]. In industrial electrochemical processes, ILs are used as green solvents for metal purification via electroplating or in the green energy sector as biofuels [49,50].

Indeed, ILs containing anions such as [BF_4_^−^], [SbF_6_^−^], and [PF_6_^−^] are generally stable in air, although the anion [PF_6_^−^] in the presence of moisture can hydrolyze producing small amounts of HF, while perfluorinated anions such as [Tf_2_N^−^] or [TfO^−^] form liquid salts with a lower viscosity combined with a higher thermal and electrochemical stability.

Indeed, the combination of CNT bucky gel with IL allows the 3D agglomerates and bundles arising from the mutual Van der Waals interactions of individual nanotubes to be prevented. To make our gels, SWCNTs enriched in chirality (7,6) from SouthWest NanoTechnologies (SWeNT SG76) with semiconductive behavior were dispersed in imidazolium-based ionic liquids, and then applied onto Si NW arrays for the realization of IL-CNT/NW hybrid platforms for energy production. This approach for a photo-responsive device realization is economical, fast, and industrially compatible. All the steps of the manufacturing process and the detailed structural characterizations are reported below, showing how the Si NWs array was well embedded in the carbon nanotubes gel. Finally, we show that the junctions obtained with this approach have promising potential for photovoltaic applications.

## 2. Results and Discussion

The core of the IL-CNT/NW hybrid cell was made up of doped p-type NWs embedded in a shell composed of a carbon nanotube and an ionic liquid gel, also called bucky. The fabrication steps of the IL-CNT/NW hybrid junction were as follows: (i) fabrication of Si NW arrays; (ii) fabrication of the electrodes by gold sputtering; (iii) preparation of the bucky gels containing the carbon nanotubes dispersed in ionic liquids and (iv) the realization of the IL-CNT/NW hybrid cell by infiltrating the CNT bucky gel into the Si NW arrays.

### 2.1. Fabrication of Si NW Arrays

**Figure 1:**
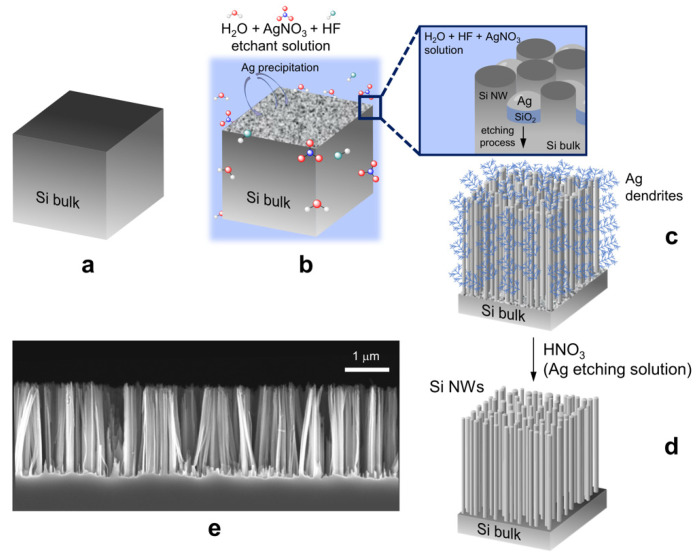
Scheme for the realization of vertical arrays of Si nanowires by Ag-mediated metal-assisted chemical etching. (**a**) The Si wafer was first treated for the removal of the native oxide and then (**b**) etched in an AgNO_3_/HF solution as described in the Materials and Methods section. (**c**) As shown in the inset of (**b**), during the etching (**c**) the formation of Ag dendrites occurred as a byproduct of the rection, (**d**) which were subsequentially removed by etching in an HNO_3_ solution. (**e**) Cross-section scanning electron microscopy of the used 2 µm long Si NW array.

In the first stage, the Si wafer was exposed to UV-ozone treatment for 5 min and then etched in a 5% HF solution for 5 min to remove the native oxide passivation (Figure 1a). Vertical arrays of Si NWs were then fabricated by metal-assisted chemical etching using an aqueous solution of silver salts and hydrofluoric acid AgNO3 (0.02 N) and HF (5 M) [51]. During the process, silver agglomerates were made into nanoclusters which were uniformly distributed on the surface of the Si wafer (Figure 1b). The electrode-less silver deposition was performed by simple galvanic displacement reactions, arising from the Si oxidation and Ag^+^ reduction, as follows:

Ag^+^ + e^−^ → Ag

Si + 2H_2_O → SiO_2_ +4H^+^ + 4e^−^

The cathodic reduction of Ag^+^ led to the clustering of metal Ag seeds onto the Si surface. The anodic oxidation of Si released electrons that were transferred to the sites of silver deposition. Then, the Ag nanoclusters acted as local cathodes at the Si interface, acting as the anode in the electrochemical redox reaction. As a result, at the Ag/Si interface the Si wafer was locally oxidized, producing SiO_2_ which was selectively etched from HF:

SiO_2_ + 6HF → H_2_SiF_6_ + 2H_2_O

Hence, as the Ag nanoclusters sank into the Si wafer, cycling the local Si oxidation and its subsequent etching, both vertically aligned Si nanowires and silver dendritic films were formed as byproduct (Figure 1c) [52,53]. After the etching process, the Si samples were rinsed with deionized water and immersed in a solution of HNO_3_ for 10 min to remove the Ag dendrites (Figure 1d). All the soakings were conducted at room temperature. The morphology of the samples was observed by scanning electron microscopy, reporting the realization of a dense array of about 2 µm long Si NWs, with an average diameter of 50 nm (Figure 1e) [54].

Once the NW array was made, a gold film with a thickness of 200 nm was deposited on the back of the NW sample through a sputtering process at a current of 50 mA for 10 min with an Emitech K500 Sputter coater under vacuum pressure of 2 × 10^−2^ mbar. Subsequentially, the IL-CNT bucky gels were prepared as described below.

### 2.2. Realization of IL-CNT Bucky Gels

The possibility of having metal-type and semiconductor-type nanotubes combined with a variety of energy gaps is very attractive for optoelectronic properties. However, individual nanotubes mutually interact with each other due to Van der Waals forces, forming 3D agglomerates and bundles which limit their practical applications. Hence, it is necessary to efficiently disperse the tangles of the CNTs to foster their infiltration into the small NW array interstices to exploit their properties [38]. The particular structure of these imidazolium-based ionic liquids allows the CNTs to untangle due to the IL rings which exert electrostatic interactions with the CNT aromatic rings. Hence, the use of ILs allows the effective screening of the electrostatic interactions between the different CNTs responsible for their skeins compared to other solvents [43,55]. For this reason, we exploited the use of debundled CNTs for an innovative hybrid cell based on Si NWs and gels of carbon nanotubes dispersed in room temperature ionic liquids, pure organic salts made solely of ions. In this work, three types of CNT bucky gels, gelatinous composites consisting of carbon nanotubes, and room temperature ionic liquids based on the imidazolium were obtained dispersing SWCNTs enriched in chirality (7,6) in the ionic liquids specified in Table 1.

For the efficient debundling of the CNT and the bucky gel formation, the following ILs all made of a 3-butyl-1-methyl-1H-Imidazolium [BMIM]^+^ anion, an I^−^ iodide cation for the BMIM-I, a BF_4_^−^ tetrafluoroborate cation for the BMIM-BF_4_, and a bis(trifluoromethanesulfonyl)imide Tf_2_N^−^ cation for the BMIM-Tf_2_N were used. The ionic conductivity is reported in Table 1 for all the adopted ILs at the environmental condition of 300 K. ILs have chemical-physical properties that can be selected as needed, such as a wide liquid range, a low flammability, a low toxicity, and a high thermal stability, which make them, as mentioned above, green solvents. All the chosen ILs have a melting point well below 300 K and do not evaporate even in ultra-high vacuum conditions. Among the three different ILs, the BMIM-I was the densest while the BMIM-BF_4_^−^ showed a higher ionic conduction. The viscosity of ionic liquids is a key feature associated with the mobility of the ions; hence, their charge transport capacity. A viscosity variation can indeed influence their transport properties of conductivity, diffusion coefficient, and charge transfer rate [56]. As for all electrolytes, the conductivity of ILs originates from the motility of the cations and anions under the application of an electric potential gradient. In our case, to achieve a uniform coverage of the dense array into the Si NW forest, we targeted a low viscosity medium that could still guarantee good conduction. Hence, since the cationic structure has a remarkable influence on IL conductivities, we opted for the choice of imidazolium-based ILs since they had a good compromise of stability, viscosity, and conductivity compared to the others available. Moreover, compared to other bile salts, micelles, or polymers used to reduce the CNT bundling, imidazolium-based ILs present an aromatic ring able to interact without π–cation interaction. In the presence of the shear force of the ILs, the bundles detach to provide individualized CNT or smaller bundles. Once the CNTs are detached, they are immediately surrounded by ionic liquids with high dielectric constants which shield the CNTs, preventing them from rebundling [57]. In the end, the isolated SWCNTs are enveloped by the ionic liquid via CNT-IL Van der Waals forces. Hence, with a simple mechanical milling, CNTs can be easily dispersed in the imidazolium-based room-temperature ionic liquids forming a thermally stable gel with a high CNT concentration.

**Figure 2:**
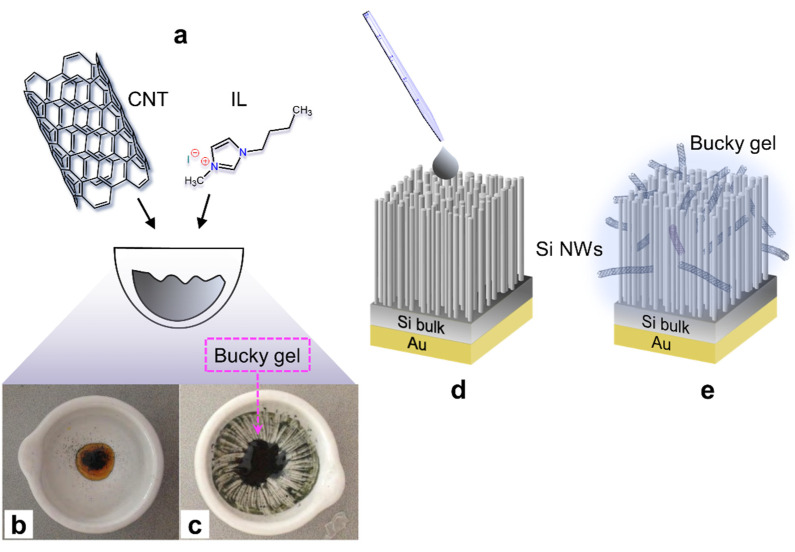
(**a**) Schematic illustration for the bucky gel realization (**b**) mixing 5.7 mg of SWCNT into 1 mL of ionic liquid into an agate mortar to be pestered for 10 min until (**c**) gel formation. (**d**) The bucky gel is drop casted onto the Si NWs array with a metal back contact to form (**e**) the hybrid IL-CNT/NW platform after its full coverage in the bucky gel.

A schematic illustration of the manufacturing steps of the coaxial device of p-doped Si NWs (100) coated with the IL-CNT bucky gel is shown in Figure 2.

**Figure 3:**
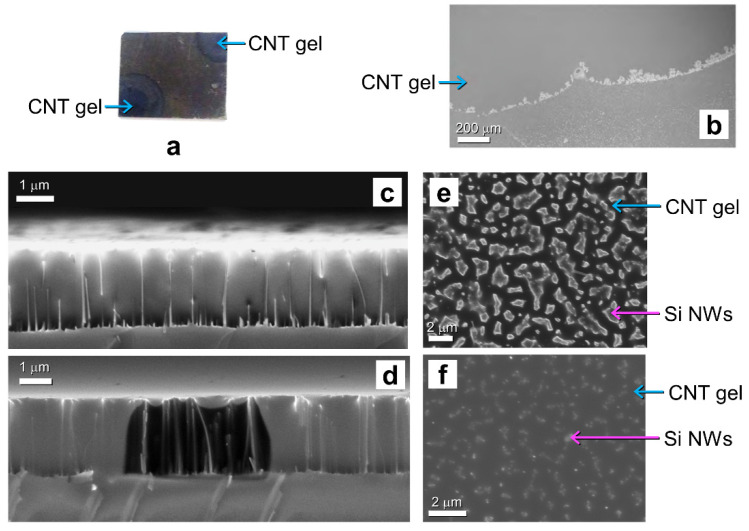
(**a**) Photo of the Si NW sample right after the application of the CNT gel at the two opposite corners. The NW sample in the photo had a dimension of 1 × 1 cm^2^. (**b**) Plan-view scanning electron microscopy during the diffusion of the CNT gel across the NW network. Cross-section SEM image of the hybrid CNT/NW platform (**c**) before and after (**d**) electron beam exposure. Plan view SEM image at the (**e**) beginning of the CNT gel diffusion process and (**f**) after 10 min, respectively.

**Figure 4:**
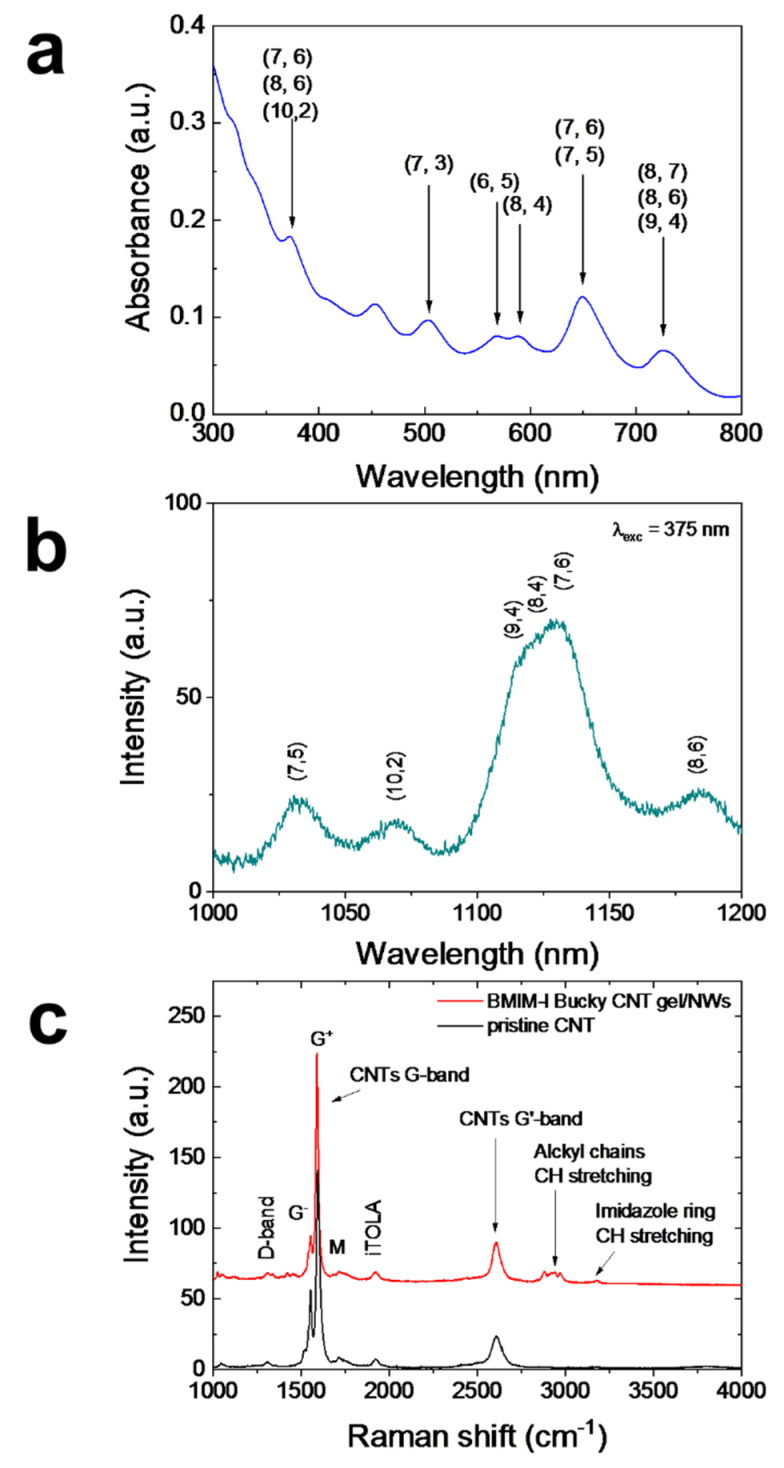
(**a**) UV-VIS absorption spectrum and (**b**) room temperature photoluminescence at 375 nm excitation spectra of the BMIM-I CNT Bucky gel with a concentration of 5.7 mg/mL. (**c**) Raman spectra of pristine CNT enriched in chirality (7,6) in black and the hybrid platform of CNT bucky BMIM-I gel applied onto the NWs in red, reported in the 1000–4000 cm^−1^ window of interest for the organic C-based bonds. The upper inset shows the SEM image of the NWs covered with the CNT gel.

As sketched in Figure 2a, for all the dispersions a mixture of 1 mL of ionic liquid (BMIM-I, BMIM-BF_4_, BMIM-Tf_2_N) and 5.7 mg of SWCNT (7,6) was pounded into an agate mortar for 10 min to form a very dense gel, as shown in Figure 2b,c. The bucky gel was then collected into an eppendorf and sonicated for 1 h. The processing time of the materials determines the viscosity of the final compound: the longer the mixture is pounded, the denser the final product will be. Subsequently, 60 µL of bucky gel was applied through a pipette onto the corners of the Si NW substrate with the Au back contact to form the hybrid IL-CNT/NW hybrid cell (Figure 2d,e). The inset in Figure 2d shows a plan view of the scanning electron microscopy of the CNT bucky gel onto the surface of the Si NWs. The formation of the IL-CNT/NW hybrid cell and the presence of the CNT gel in the ionic liquid were verified by SEM and Raman analyses, as demonstrated in Figure 3 and Figure 4, respectively.

The evolution of the infiltration process in the dense network of NWs was monitored by SEM analysis, verifying that the NWs were completely incorporated into the CNT bucky gel for all the adopted ionic liquids. Once prepared, the CNT bucky was deposited onto the two opposite corners of the NW sample as shown in the photo in Figure 3a. The presence of the spotted CNT gel was clearly visible and highlighted with the light blue arrows onto the NWs sample with a dimension of 1 × 1 cm^2^. The applied gel quickly diffused from the opposite corners across the NWs network, wrapping them completely. As a confirmation of the improved absorption of Si NWs, it is worth noticing the black appearance of the Si NWs corresponding to a reduced reflectance below the value of 10% in the visible range. The diffusion process was analyzed by SEM, and the evolution of the drop was observed at different time intervals. At the top of Figure 3b, it is possible to see the edge front of the bucky gel diffusing across the NWs. In the lower part of the SEM image, the NWs appeared as a series of brighter points not yet covered by the gel. The interface region was also brighter, showing the CNTs crowding at the drop front as it diffused. The NWs behaved similar to a sponge; in fact, the gel spread from the sides towards the center until it completely covered and incorporated the NWs. The gel diffusion was investigated both in cross-sections and (Figure 3c,d) and plan views (Figure 3e,f) right after its application, as shown in Figure 3c,e, and then after 10 min (Figure 3d,f). Further proof of the realization of the core-shell structure consisting of NWs incorporated in the IL-CNT gel is reported in Figure 3c showing the SEM cross-section after its infiltration. In Figure 3c we can clearly observe the gel completely wrapping the NWs, whose length is barely visible. After 10 min of e-beam exposure, the gel diffused laterally leaving a hole in the gel matrix that showed without any ambiguity the presence of NWs (Figure 3d). We also observed that the hole could be refilled with the gel after a few minutes after the beam exposure. As shown in the previous SEM image, the NWs immersed in the gel were barely visible when not subjected to the beam focalized exposure. The comparison of the beam exposed area with the neighboring unexposed regions demonstrates the complete coating of the NWs in the CNT gel. In Figure 3e,f, it is possible to see the SEM plan-view images acquired at the same point after 10 min. At the beginning of the gel diffusion, the contours of the NWs appeared broadened and blurred due to the presence of the gel (Figure 3e). Repeating the SEM scan in the same point of Figure 2e after about 10 min, it is visible that the tips of the NWs were completely covered by the gel (Figure 3f). By comparing the two plan views, it can be seen that the gel diffused rapidly from the sample edges towards the center, diffusing across the NW holes from the bottom first and then wrapping upwards onto the tips until their complete incorporation into the CNT gel.

The UV-VIS spectrum in Figure 4a shows a series of absorption peaks characteristic of the many CNT chiralities present in the bucky gel [58,59]. The major limitation of the use of CNT is indeed the formation of bundles due to van der Waals interactions between neighboring tubes that heavily affect their PL due to the occurrence of energy transfer among adjacent tubes arising from π–π interactions. Since each bundle may contain both semiconducting and metallic CNTs, when energy transfer to metallic tubes occurs, light emission is quenched [60]. Sharp PL peaks can be only observed in isolated nanotubes or in small bundles, in which non-radiative decay channels occurring via exciton energy transfer towards metallic species are minimized [34]. Room-temperature optical emission of semiconducting SWCNTs in the IR range is associated with the excitonic radiative recombination from the first excited levels (eh11) towards the ground state [35,61]. The PL spectrum of the bucky gel under optical excitation at 375 nm in Figure 4b shows the resonant emission from the eh33 transition which non-radiatively decayed towards the eh11 and radiatively decayed to the ground state for the (7,6) family at the wavelength of about 1130 nm, together with the other chirality emission peaks. Moreover, both the absorption and emission peaks were quite sharp, demonstrating that the adopted dispersion in ionic liquid avoided the formation of CNT bundles. The Raman measurements of the CNT bucky gels diffused into the NWs were carried out to verify the effectiveness of the carbon nanotubes debundling into the ionic liquid. The Raman spectrum of the BMIM-I CNT/NWs hybrid platform is shown in Figure 4c (red line) as representative of all the samples and compared to the spectrum of the pristine CNT powder (black line). Both spectra show the same characteristics peaks aside from the region of the CH-stretching and imidazolium ring vibrations due to the imidazolium ionic liquid, demonstrating that the electronic structure of SWCNTs in the bucky gel remained unchanged. From the Raman spectra, it is possible to notice that the characteristic CNT G-band consisting of G^-^ and G^+^ contributions peaked at about 1560 and 1590 cm^−1^, respectively [62]. The G^-^ feature was due to the in-plane stretching vibrations of C atoms along the circumferential direction, generally with a lower intensity for CNTs with smaller diameters and with a strong and asymmetric peak for metallic CNTs, while the second peak G^+^ was due to in-plane vibrations along the axis of the tube. As expected, the Lorentian shapes for both the G^−^ and G^+^ bands highlight the prevalence of semiconducting CNTs in our case [63]. It is also possible to notice in Figure 4 the presence of a small D-band at 1307 cm^−1^ associated with the disorder of the CNT lattice. Moreover, the peak at 2604 cm^−1^ of its overtone, the G’-band, was present [64]. Further vibrations coming from semiconducting tubes can be observed, such as the M and iTOLA bands at 1730 cm^−1^ and the at 1927 cm^−1^ respectively, the latter being a combination of optical and acoustic modes [63]. Moreover, the CH-stretching vibrations of the imidazolium-based ionic liquid were visible in the 2800–3050 cm^−1^ range (alkylic tail vibrations) and 3075–3250 cm^−1^ one (imidazolium ring) for the CNT bucky only (red spectrum) [65].

**Figure 5:**
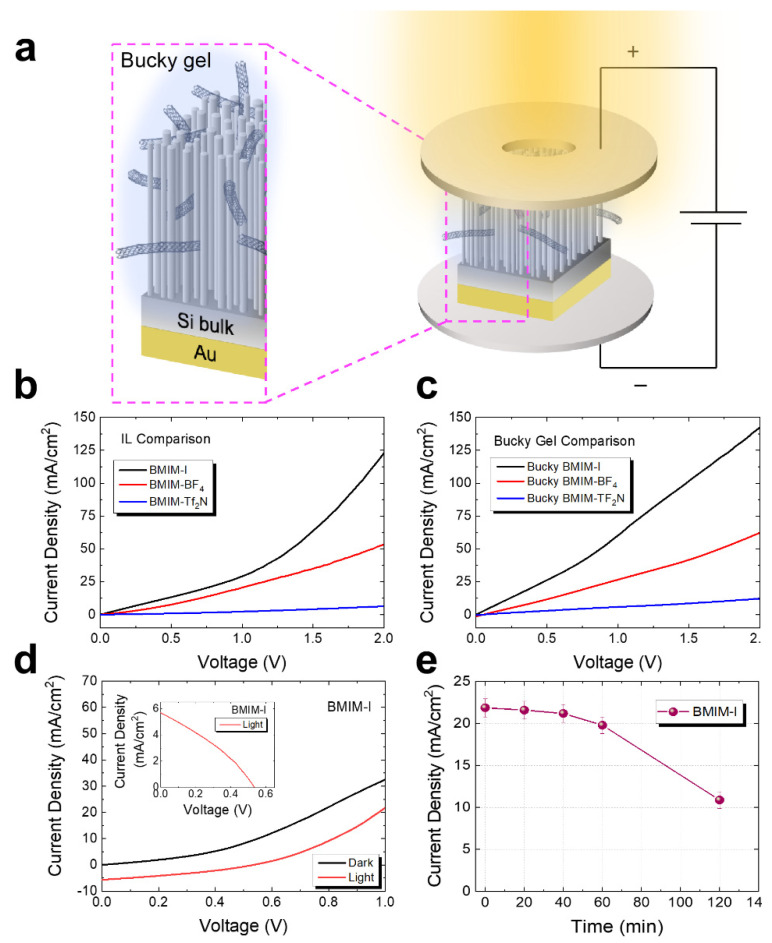
(**a**) Scheme depicting the realization of the hybrid CNT/NW platform and its electrical characterization in a sealed cell under dark and illumination conditions. The current densities are reported for the BMIM-I (black line), BMIM-BF_4_ (red line), and BMIM-TF_2_N (blue line) and (**b**) simple ionic liquids and (**c**) CNT bucky gel without the NWs for comparison. (**d**) Current density for the hybrid CNT/NW platform based on a BMIM-I matrix under dark (black line) and illumination conditions (red line). (**e**) Time stability of the photovoltaic response of the hybrid BMIM-I CNT/NW platform at a bias of 1 V under illumination at a power of 100 mW/cm^2^.

Finally, the current density–voltage measurements (J-V) with and without illumination were carried out to demonstrate the potential of these IL-CNT/NW hybrid cells for photovoltaic applications. Once the preparation of the core-shell junction consisting of p-doped Si NWs and IL-CNT gel was completed, the electrical characterizations were performed for all samples. As depicted in the scheme reported in Figure 5, the core-shell hybrid p-n junction was biased with a sealed homemade cell for the electrical and photoconductivity measurements. The Au-covered back of the Si NWs sample was lodged onto the copper rear electrode with a specific well for the hybrid NWs sample positioning and was laterally sealed [12]. The top electrode instead was made of an internal o-ring cavity wrapping the NWs and preventing the lateral gel loss. The top copper electrode presented a hole to perform the electrical measurements both under dark or illumination conditions with a Xe solar lamp. It is worth noticing that room-temperature ionic liquids have negligible vapor pressures and do not evaporate under environmental conditions. As depicted in the scheme of Figure 5a, the top and rear electrodes were externally biased with a Keithley multimeter.

Figure 5b shows the comparison of the current densities of the ionic liquids onto the Si NWs in the absence of the CNTs measured as a function of the applied bias for the BMIM-I (black line), BMIM-BF_4_ (red line), and BMIM-TF_2_N (blue line), respectively.

The comparison attests that the BMIM-I/Si NW hybrid cell had the highest current density about 20 times the value of the BMIM-TF_2_N and 2.3 times the BMIM-BF_4_ at the voltage of 2 V, possibly due to the high mobility of the very small iodide ions compared to the other IL ions. When the bucky gel was formed in presence of the CNTs, the J-V of the hybrid CNT gel/NWs junctions confirmed that the BMIM-I CNT gel still showed the best electrical performance, as reported in Figure 5c. This can be ascribed to the increment of the BMIM-I matrix conduction due to the presence of the CNTs, while in the presence of the other gel no significant variation of the J-V curve was observed, hence confirming the debundling efficiency in the BMIM-I ionic liquid compared to the rest. We then focused our attention on the BMIM-I CNT gel/NWs hybrid cell, which possessed the best electrical performance, to test its photo-response as shown in Figure 5d in dark and under illumination with a Xe Lamp solar simulator at a power of 100 mW/cm^2^ (AM 1). The black line shows the current–density trend in the absence of lighting (dark condition), and we measured current densities of the order of about 33 mA/cm^2^ at a bias of 1 V for the hybrid cell of 1 × 1 cm^2^. The red curve indicates the variation of current density under solar simulator illumination with a power density of about 100 mW/cm^2^, reporting an overall decrease in the J-V trend of about 0.63 compared to the dark condition.

The trends shown in Figure 5d demonstrate the potential of the BMIM-I CNT/NWs hybrid coaxial junctions for photovoltaic applications. In the inset of Figure 5d, we reported the modulus of the current density (|-J|) as a function of the applied bias to measure the photovoltaic characteristics of the cell with a short-circuit current density (J_SC_) and open-circuit voltage (V_OC_). From the analysis we obtained the following values: J_SC_ = 5.6 mA/cm^2^ and V_OC_ = 0.53 V. For the BMIM-I CNT/NWs, we measured a filling factor of about 0.33, and about 0.28 and 0.25 for the BMIM-BF4 CNT/NWs and the BMIM-TF_2_N CNT/NWs, respectively. For all samples we observed the presence of both shunt and series resistance, as can be noticed in the inset of Figure 5d, possibly induced by our homemade system.

Finally, we also considered the stability of the photovoltaic response for 2 h (Figure 5e) for the hybrid BMIM-I CNT/NW platform at a bias of 1 V, attesting that the cell remained quite stable below a variation of 12% for 1 h, undergoing a 50% decrease in the current density after 2 h of operation, which could be ascribed to the hygroscopic behavior of the ionic liquids. In this study, we have presented a preliminary analysis that would allow taking advantage of the whole vertical array of Si nanowires compared to the planar structure used in commercial Si PV cells. Si NWs provide a natural surface texturing effect without the use of expensive processes leading to a strong suppression of the device reflectance [66]. This would improve the light scattering and, in the end, the charge production. Additionally, in order to efficiently collect the light-induced charge carriers in a 3D structure such as our vertical array of Si NWs, we investigated the radial charge extraction in the bucky gel. Indeed, due to the narrow spacing among adjacent NWs is it hardly possible, without expensive processes such as atomic layer deposition, to perform it with a uniform continuous film on the NW dense array which is 3 µm long. Hence, it is more interesting to use a liquid media to extract and collect the charges produced in the NWs from the solar illumination.

## 3. Materials and Methods

*Si Nanowires:* Si NWs were obtained from the metal-assisted chemical etching of Si wafers (Sigert Wafer GmbH, Germany) with silver nitride (0.02 N from Carlo Erba, Conaredo, Milano, Italy) and hydrofluoric acid (50% from Sigma Aldrich, Steinheim am Albuch, Baden-Württemberg Germany, Europe). The Ag dendrites contaminants were removed by nitric acid (90% reagent grade from Carlo Erba, Conaredo, Milano, Italy).

*Carbon Nanotubes:* commercially available single-walled CNTs enriched in semiconductive chirality (7,6) were purchased from SouthWest NanoTechnologies, Norman, OK, USA, (SWeNT SG76, Lot #SG76-L29).

*Ionic Liquids:* 1-Butyl-3-methylimidazolium iodide (BMIM-I, 98%), 1-Butyl-3-methylimidazolium tetrafluoroborate (BMIM-BF_4_, 99%), and 1-Butyl-3-methylimidazolium bis(trifluoromethanesulfonyl)imide (BMIM-Tf_2_N, 99%) were all acquired from IoLiTec-Ionic Liquids Technologies GmbH, Zukunftspark 9 D-74076 Heilbronn Deutschland.

*Raman Measurements:* Raman spectra were performed with a micro-Raman HR800 system (Jobin-Yvon, Horiba, Duwijckstraat 17, 2500 Lier, Belgium) with a 100× objective with a 633 He-Ne laser at a power of about 100 µW.

*Optical Measurements:* UV-VIS absorbance measurements were performed with a PerkinElmer Lambda 20 spectrometer, while the photoluminescence spectrum was acquired with a NanoLog spectrofluorimeter equipped with a Xe lamp, and an InGaAs N_2_-cooled detector from Horiba Jobin-Yvon.

*Scanning Electron Microscopy:* SEM images were obtained at a working voltage of 3 kV with an InLens detector by using a Gemini Field ZEISS (Carl-Zeiss-Straße 22, 73447, Oberkochen, Germany) Electron Microscope equipped with a field-effect e-source.

*Electrical characterizations:* current density measurements were performed at room temperature with a Keithley (9990 Fairfax Boulevard, Suite 340, Fairfax, VA 22030, US) 2002 multimeter under dark or light conditions at 100 mW/cm^2^ using an Oriel solar simulator equipped with a Xe lamp.

## 4. Conclusions

In conclusion, this study demonstrated the realization of hybrid coaxial junctions based on a core of Si NWs embedded into three different types of bucky gels composed of carbon nanotubes well dispersed in an imidazolium-base ionic liquid matrix. We reported the structural and morphological properties of the hybrid systems and demonstrated the uniform diffusion of the bucky gel inside the NWs. Moreover, we investigated the electrical response of the hybrid junctions in dark and under illumination for three different bucky gels and we correlated the results with ionic liquid characteristics and charge mobility. The reported results demonstrate the potentialities of this low-cost and green hybrid system for energy applications.

## Figures and Tables

**Table 1:** List of the 3 types of adopted room temperature ionic liquids used for the realization of the CNT bucky gels.

Ionic Liquid	Ionic Conductivity @ 300 K	Viscosity @ 300 K	Bucky Gel ^1^
1-Butyl-3-methylimidazolium Iodide (**BMIM-I**)	0.517 mS/cm	1187 mPa·s	Bucky BMIM-I
1-Butyl-3-methylimidazolium tetrafluoroborate (**BMIM-BF_4_**)	3.15 mS/cm	103 mPa·s	Bucky BMIM-BF4
1-Butyl-3-methylimidazolium bis(trifluoromethanesulfonyl)imide (**BMIM-Tf_2_N**)	0.6 mS/cm	350 mPa·s	Bucky BMIM-Tf2N

^1^ For all cases, 1 mL of ionic liquid was mixed with 5.7 mg of SWCNT enriched in chirality (7,6). For all the adopted ILs, the melting temperature was well below room temperature.

## Data Availability

Not applicable.
